# Thymol

**Published:** 1878-08

**Authors:** 


### ARTICLE VI.

### *Thymol.*

The essential oils of thyme, of American horsemint, and of the *Ptychotis ajowan* contain a substance, a homologue of phenol or carbolic acid, having the composition represented by $C_{10}H_{14}O$, and known as thymol. For more than two years this has been used by German surgeons, and is now being introduced among ourselves. It was discovered in 1719 by Caspar Neumann, examined chemically by Lallemand and Leonard Doveri, and first used to deodorise unhealthy wounds by Bouillon and Paquet, of Lille, in 1868. In 1875 several German surgeons published investi gations of its antiseptic properties, which are estimated to be from 4 to 25 times as powerful under certain circumstances as those of carbolic acid. Thymol is a crystalline, nearly colourless body, with a pleasant odour and an aromatic burning taste. Its specific gravity is 1.028, and it melts at 44° C. It dissolves in 1,200 parts of cold water, 1 part of rectified spirit, 120 parts glycerine, and in $\frac{1}{3}$ part of caustic alkalies. Fats and oils also dissolve it readily. It is prepared from the oils of either of the plants before mentioned, but pharmacists should beware of experimenting on English samples of oil of thyme, as but few of them are genuine, or, at least, contain any thymol. The oil is said to yield as much as 50 per cent. of thymol on the Continent. Thymol can be manufactured from these oils by treating them with an equal volume of a 20 per cent. solution of caustic soda, separating the alkaline liquid, and neutralising with hydrochloric acid, when the thymol will float to the surface. It may also be obtained by submitting the oils to a low temperature for a few days, when the thymol crystallises out. Its powerful antiseptic action, exceeding under some conditions that of carbolic acid, its small activity as a poison—about one-tenth of that of carbolic acid—and the absence of irritating effect when it is applied to the skin, all point to its use as a substitute for carbolic acid in the now well-known antiseptic treatment of surgical cases elaborated by Professor Lister. This substitution has been made with great success by Professor Volkmann, of Halle. For the spray solution, this gentleman uses a mixture of 1 part thymol, 10 alcohol, 20 glycerine, 1,000 water; but we understand that a solution in water only, which will not deposit, may be made by adding 1 part of thymol to 1,000

of hot water. For the gauze dressings used by Professor Lister, others were substituted, made by saturating 1,000 parts of bleached gauze with a mixture of 500 parts spermaceti, 50 resin, and 16 of thymol. This prepared gauze is extremely soft and pliant, and, to use the words of the reporter, sucks up blood and the secretions of the wound like a sponge. The fibres of the gauze being impregnated with spermaceti, cannot, of course, become saturated with the secretions, so that they do not become stiff. Thymol has been used for various skin diseases by Dr. R. Crocker, but the results of his experiments have not yet been published. As an internal remedy, thymol does not seem to make much way. It has proved useful in diseases of the stomach accompained by fermentation, and Mr. W. H. Stone reports in the *Medical Times and Gazette* that he has found it useful in cases of chorea, one form of which is St. Vitus' Dance. The present cost of thymol is about five times that of the best carbolic acid, but as one part of the former seems to do as much work as 25 parts of the latter, the advantage of price is on the side of thymol.—*Chemist and Druggist.*

---

